# The Impact of a Telehealth Intervention on Activity Profiles in Older Adults during the COVID-19 Pandemic: A Pilot Study

**DOI:** 10.3390/geriatrics6030068

**Published:** 2021-06-30

**Authors:** Nathaniel Johnson, Adam Bradley, Lukus Klawitter, Jane Johnson, Lance Johnson, Grant R. Tomkinson, Kyle J. Hackney, Sherri Stastny, Diane K. Ehlers, Ryan McGrath

**Affiliations:** 1 Department of Health, Nutrition, and Exercise Sciences, North Dakota State University, Fargo, ND 58108, USA; nathaniel.johnson.4@ndsu.edu (N.J.); adam.bradley@ndsu.edu (A.B.); lukus.klawitter@ndsu.edu (L.K.); janepjohnson@gmail.com (J.J.); lance5000johnson@gmail.com (L.J.); kyle.hackney@ndsu.edu (K.J.H.); sherri.stastny@ndsu.edu (S.S.); 2 Department of Education, Health and Behavior Studies, University of North Dakota, Grand Forks, ND 58202, USA; grant.tomkinson@und.edu; 3 Alliance for Research in Exercise, Nutrition and Activity, School of Health Sciences, University of South Australia, Adelaide, SA 5501, Australia; 4 Department of Neurological Sciences, University of Nebraska Medical Center, Omaha, NE 68198, USA; diane.ehlers@unmc.edu; 5 Fargo VA Healthcare System, Fargo, ND 58102, USA

**Keywords:** aging, computers, exercise, geriatrics, internet, movement, telemedicine

## Abstract

Background: Physical inactivity during the COVID-19 pandemic is a public health concern for older adults. Telehealth presents a safe platform for conducting health-related interventions that may have additional benefits such as widespread reach. Our pilot study sought to examine how a telehealth intervention changed activity profiles in older adults during the COVID-19 pandemic. Methods: There were *n* = 13 adults aged 70.6 ± 4.5 years that participated in a 6 week telehealth intervention during the COVID-19 pandemic. The didactic intervention contents were shared online, and participants worked with trained interviewers over the telephone to discuss physical activity. At baseline and post-intervention, the Multimedia Activity Recall for Children and Adults examined activity profiles, while accelerometry estimated time spent sedentary and in physical activity. Results: Relative to the baseline measures, there was an 88 min/day (95% confidence interval (CI): 39, 137) increase in computer time and 36 min/day (CI: 10, 62) reduction in time spent in active transport at post-intervention. Moderate-to-vigorous physical activity participation also increased by an estimated 2 min/day (CI: −21, 26) and 12 min/week (CI: −154, 180), but this trend was not statistically significant. Conclusion: We recommend that support be provided to older adults transitioning to telehealth, especially as migration to telehealth progresses.

## 1. Introduction

Healthy lifestyle behaviors such as physical activity participation are robustly associated with successful aging and delayed all-cause mortality [1,2]. However, approximately two-thirds of older adults are sedentary for more than 8.5 h/day [3]. The COVID-19 pandemic may have especially exacerbated physical inactivity in older adults. For example, older adults engaged in higher sedentary behaviors, lower physical activity participation, and reported a decline in their physical health during the COVID-19 pandemic [4,5]. As such, interventions that help older adults safely overcome barriers to physical activity participation are urgently needed to help maintain physical health and successful aging [6].

Telehealth has emerged as a relatively inexpensive and accessible platform for performing healthy lifestyle interventions that also has widespread catchment for difficult to reach populations such as rural older adults [7]. Previous studies that have utilized telehealth for delivering wellness interventions have found that older adults valued this platform and used the internet for researching health-related information [8,9]. Moreover, the effectiveness of delivering health-related information intended for behavior change through telehealth is equal to that of print-based delivery [10]. While telehealth presents a promising platform for conducting interventions, telehealth interventions need to be further examined in older populations [11,12], particularly in light of the COVID-19 pandemic [13]. Our pilot study sought to examine how a telehealth intervention changed activity profiles in older adults during the COVID-19 pandemic.

## 2. Materials and Methods

### 2.1. Participants

This pilot investigation utilized a prospective, within-participant design, with measures before and after the 6 week intervention period. Repeating measures allowed us to examine within-participant changes over time and optimize our analyses with sample sizes that are often observed in pilot studies. Participants were recruited in February 2021 through word of mouth, flyers, online advertisements, and university email listservs. To participate in our pilot telehealth intervention, participants needed to be aged at least 65 years, cognitively intact, able to read and speak the English language fluently, able to wear an accelerometer, have a body mass index ≥18.5 kg per meters-squared (i.e., not underweight) [14], and able to engage in physical activity as determined by the PAR-Q+ [15]. 

People who were interested in our pilot intervention contacted a trained interviewer to evaluate eligibility. To maintain the telehealth platform, all study criteria were determined by self-report. Of the *n* = 16 people who contacted a trained interviewer to determine eligibility, *n* = 2 individuals were declared as ineligible, and *n* = 1 decided not to continue before the intervention began. The remaining *n* = 13 participants provided written informed consent before engaging in our pilot study and protocols were approved by the North Dakota State University Institutional Review Board (Protocol ID: IRB0003367). 

### 2.2. Intervention

This pilot study was registered on clinicaltrials.gov (NCT04461184). The telehealth intervention began in March 2021 and was 6 weeks in duration. Intervention contents were shared with participants on Google Classroom [16], with anonymous Google Classroom accounts created. Interviewers trained participants in how to effectively utilize their Google Classroom accounts to access intervention contents.

Few intervention studies in gerontology research exist where older adults have a research role [17]. Therefore, the study team included two older adult stakeholders that contributed to all phases of the telehealth intervention in order to provide meaningful perspectives for our intervention that are relevant to the older adult population we studied [18,19]. Specifically, the older adult stakeholders helped in tasks such as developing the intervention contents, quality controlling navigation in Google Classroom, piloting, troubleshooting, and disseminating this work. Contents of the intervention were guided by physical activity and other related healthy behavior recommendations for older adults [20,21,22,23]. 

During the first three weeks of the intervention, participants were asked to log into Google Classroom, at least weekly, and view didactic materials related to practicing healthy lifestyle behaviors (i.e., physical activity). The didactic materials were created by the investigators and included study-specific videos, pre-recorded presentations and demonstrations of physical activities, and readings that were germane to practicing healthy lifestyles overall and during the COVID-19 pandemic. Following the didactic portion of the telehealth intervention, trained interviewers worked individually with participants over the telephone to discuss the intervention and develop a customized strategy for practicing physical activity that aligned with each participant’s abilities and environment. Theory-based goal setting was utilized to encourage participants to practice the physical activities from the intervention [24]. Participating in light and moderate-to-vigorous physical activity was emphasized. Providing autonomy to physical activity participation, a sense of relatedness between participants and interviewers, and reinforcing competence in physical activities was encouraged [25]. 

### 2.3. Measures

At baseline, participants completed a self-reported demographic questionnaire in Google Classroom. The Multimedia Activity Recall for Children and Adults (MARCA), a computer-assisted physical activity recall, was utilized to capture each participant’s activity profiles. The MARCA asked participants to recall their previous day chronologically from midnight to midnight using meal times as anchor points in a segmented day format. Participants recalled their day in time periods of five minutes or more by choosing from over 500 discrete activities, with each activity linked to a compendium of energy expenditures [26,27]. 

The adult version of the MARCA was administered by trained interviewers on the telephone at both baseline and post-intervention, each time recalling the two previous days. This version of the MARCA has demonstrated a very high test–retest reliability (ICC = 0.92–0.99) and high validity with accelerometry (rho = 0.72) and doubly labeled water (rho = 0.70) in adults [28,29]. As outlined in Table 1, MARCA activities were hierarchically aggregated into nine super-domains with appropriate sub-domains [30]. To capture typical weekly activity patterns, the time spent in each activity domain was averaged across the two days using a 5:2 weighting for weekdays:weekend days.

Physical activity participation was monitored at baseline and post-intervention with an ActiGraph GT9X-BT accelerometer (ActiGraph; Pensacola, FL, USA). Each accelerometer was initialized at 30 Hz with ActiLife 6 software (ActiGraph) [31]. Accelerometers, wearing instructions, and logs (to record beginning and end time for physical activity, accelerometer removals, and sleep) were sent to each participant in the mail. Trained interviewers also communicated with participants over the telephone and online regarding how to wear the accelerometer and complete logs. Participants were asked to wear the accelerometer on their non-dominant wrist during waking and sleep time for seven consecutive days, and only removing the accelerometer for water-based activities (e.g., bathing, showering, swimming) [32]. Participants returned the accelerometers and logs in the mail after their requested wear time was completed. 

Data were stored in 60 s epochs [33]. Minimum wear time was defined as at least 10 h/day during waking time for four days including one weekend day [34]. The Choi et al. [35] non-wear algorithm was used to remove periods of non-wear during waking time. Time spent in sedentary behavior and each intensity of physical activity was determined with applicable cut-points: <2860 counts/min for sedentary behavior, 2860–3940 counts/min for light physical activity, and ≥3941 counts/min for moderate-to-vigorous physical activity [36]. Daily sedentary and physical activity time estimates were summed and subsequently used to determine weekly estimates. The same accelerometer protocols were used for both the baseline and post-intervention measurements. 

### 2.4. Statistical Analysis

The mean ± standard deviation number of minutes spent by participants in each activity domain was calculated at baseline and post-intervention, with the change from baseline determined. Changes in time use as determined from the MARCA were expressed as min/day. Positive changes indicated more time spent in each activity domain while negative changes indicated less time spent in each activity domain. Further, the daily and weekly mean ± standard deviation changes in time spent sedentary and in each intensity of physical activity were determined by comparing baseline estimates to those at post-intervention. SAS 9.4 software (SAS Institute; Cary, NC, USA) was used to analyze the accelerometer data. An alpha level of 0.05 was used for all analyses. 

## 3. Results

**Figure 1:**
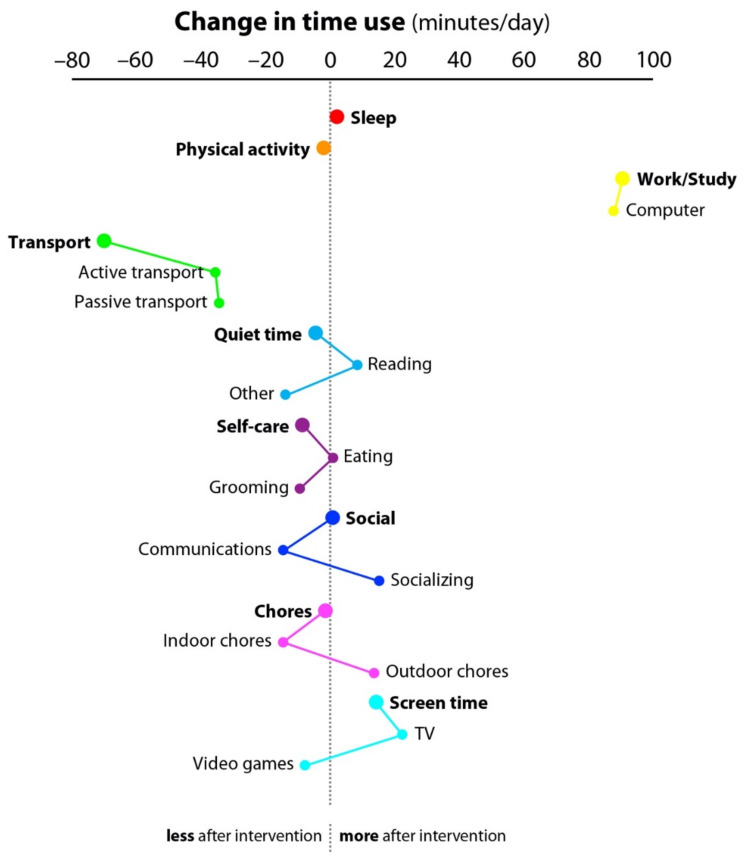
Changes in Time Usage across Activity Domains from Baseline to Post-Telehealth Intervention. Note: Dots to the right of the dashed vertical line indicate an increase in the time spent in the super- or sub-domains, and dots to the left of the dashed vertical line indicate a decrease in the time spent in the super- or sub-domains. Sub-domains corresponding to a super-domain are linked by lines. min/day = min/day.

The baseline descriptive characteristics of the *n* = 13 participants are shown in Table 2. Overall, the participants were aged 70.6 ± 4.5 years and *n* = 10 (76.9%) identified as female. All participants completed the MARCA before and after the telehealth intervention. The time participants spent in each MARCA activity super- and sub-domain at baseline and post-intervention is shown in Table 3, while the corroborating changes in time usage from baseline to post-intervention across activity domains are depicted in Figure 1. At the end of the intervention period, there was a 91 min/day (95% confidence interval (CI): 45, 136) increase in time spent in work and study, primarily because of an 88 min/day (CI: 39, 137) increase in computer time. These increases were compensated by a 36 min/day (CI: 10, 62) reduction in time spent in active transport. 

Only a single day of accelerometer wear was removed from the analyses post-intervention due to wear time adherence <10 h. The estimated mean wear time at baseline was 950 ± 52 min/day (6651 ± 365 min/week) and at post-intervention was 908 ± 69 min/day (6307 ± 584 min/week). Table 4 presents the estimated time spent in sedentary behavior and physical activity during the telehealth intervention. Although there was a non-significant trend, estimated daily and weekly moderate-to-vigorous physical activity participation increased by 2 min (CI: −21, 26) and 12 min (CI: −154, 180) after the intervention was completed, respectively. 

## 4. Discussion

This 6 week pilot telehealth intervention that occurred during the COVID-19 pandemic revealed that older adults spent approximately 88 more min/day working and studying in front of a computer, and approximately 36 less min/day engaging in active transport. Although our older adult participants reported spending more time in front of a computer and less time in active transport after the intervention was completed, a non-significant trend in participants engaging in more minutes of daily and weekly moderate-to-vigorous physical activity was observed, respectively. These findings suggest that participants may have replaced engaging in physical activity through active transport with participating in physical activity in front of a computer while using our intervention as a guide. 

Our findings add to the important and growing body of literature related to health interventions for older adults during the COVID-19 pandemic. The COVID-19 pandemic elicited unhealthy behaviors in older populations including lower physical activity participation and prolonged bouts of sedentary behavior [5]. Several building closures (e.g., exercise facilities, malls) and safety fears due to the COVID-19 pandemic may have driven physical inactivity in older adults [6], and the creation and implementation of interventions that promote safe physical activity are needed in the event subsequent emergencies occur [37]. Our pilot telehealth intervention provided a unique platform for the safe promotion of healthy lifestyles. The building closures and safety fears from the COVID-19 pandemic may have underpinned why our participants spent more time in front of a computer and less time in active transport after our telehealth intervention was completed. Interestingly, participants also spent more time engaging in moderate-to-vigorous physical activity while spending time in front of a computer post-telehealth intervention, albeit this trend was not statistically significant. 

While our pilot telehealth intervention showed a non-statistically significant trend for improving moderate-to-vigorous physical activity during the COVID-19 pandemic, other outcomes and benefits of utilizing telehealth interventions for older adults should be acknowledged. For example, older adults residing in rural areas may have limited access to health-related services due to a scarcity of services, insufficient public transport, and financial constraints [38]. Telehealth interventions provide a relatively low-cost and convenient platform that has widespread reach for such people to receive health-related services. Telehealth could also be used to complement face-to-face interventions [39]. The large and potentially permanent migration to telehealth as a result of the COVID-19 pandemic should underscore the need to support older adults in using relevant technologies and providing necessary resources [40]. Investing in such resources could be especially critical given that the older American population is projected to grow approximately 112% by the year 2060 [41]. 

Some study limitations should be noted. Although our sample size exceeds the minimum recommended amount for single-group pilot studies [42], our ability to conduct more robust analyses was limited due to sampling. While we wanted to maintain intervention simplicity during the COVID-19 pandemic for our older adult participants, follow-up data after intervention completion were not available and therefore sustainable physical activity behavior change could not be observed from our pilot study. Likewise, examining sex as a biological variable with sex-stratified analyses was not completed because of our pilot-level sample size. Convenience sampling was applicable to the recruitment of our participants. This telehealth intervention took place during the COVID-19 pandemic, and health-related behaviors during this time may not reflect such behaviors before the pandemic began. Similarly, our telehealth intervention may have elicited a Hawthorne effect on participants, and thus any changes in physical activity profiles could be attributed to the Hawthorne effect instead of the intervention itself. Our participants all identified as non-Hispanic white and were well educated. Therefore, generalizability of our findings is limited. 

Future research should continue investigating the utility and efficacy of telehealth interventions in older populations, especially for those living with muscle dysfunction and morbidities that can undermine independence such as obesity. Larger sample sizes, stronger experimental designs with follow up and control groups, and additional objective measures should also be considered for this translational research when possible. Given that our participants were overall compliant with our intervention, factors such as maintaining routine communication with participants, including older adult stakeholders on the study team, and personalizing activity recommendations may help to preserve participant engagement. Continuing to monitor telehealth participant recruitment, satisfaction, feasibility (participant and investigator), and outcome efficacy in different populations will help to determine the utility of telehealth interventions, especially for older people with lower computer access and competency. Providing more computer access and didactic computer use sessions for applicable older adults as a study aim may help to streamline telehealth as an intervention mode.

## 5. Conclusions

This pilot telehealth intervention which occurred during the COVID-19 pandemic revealed that older adult participants spent more time on a computer and less time in active transport after the intervention was completed. It is also possible that participants were utilizing our telehealth intervention contents to engage in moderate-to-vigorous physical activity while in front of the computer. Continued research for telehealth interventions is warranted, especially as migration to telehealth progresses. We recommend that support be provided to older adults transitioning to telehealth, especially after the COVID-19 pandemic. 

## Figures and Tables

**Table 1:** Activity Super- and Sub-Domains as Determined from the Multimedia Activity Recall for Children and Adults.

Super-Domain	Sub-Domain	Description	Examples
Sleep	-	Sleeping, napping	Day time nap
Physical Activity	-	Formal and informal exercise	Gym, sports
Work and Study	-	Occupational activity, study	Office work
-	Computer	Non-gaming computer use	Internet, email
Transport	-	Locomotion	Driving a car
-	Active transport	Non-motorized transport	Cycling, walking
-	Passive transport	Motorized transport	Riding a bus
Quiet Time	-	Non-social leisure time	Listen to music
-	Reading	Recreational reading	Reading an article
-	Other	Non-reading quiet time	Lying in bed
Self-Care	-	Eating, grooming	Eating dinner
-	Eating	Eating or drinking	Drinking coffee
-	Grooming	Grooming or ablutions	Showering
Social	-	Social and cultural activities	Talking
-	Communications	Communicating with others	Calls, text message
-	Socializing	Social interactions	Parties
Chores	-	Domestic tasks	Food prep
-	Indoor chores	Inside tasks	Vacuuming
-	Outdoor chores	Outdoor tasks	Gardening
Screen Time	-	Phone apps, television	Phone apps
-	Television or videogames	Watching television, movies	Watching movies

Note: Activity domains are defined using lists of activities, and the rubrics are only indicative.

**Table 2:** Descriptive Characteristics of the Participants.

Variable	Value
Age (Years)	70.6 ± 4.5
Female (*n* (%))	10 (76.9)
Non-Hispanic White (*n* (%))	13 (100.0)
Completed Bachelor’s Degree or Higher (*n* (%))	9 (69.2)
Retired (*n* (%))	12 (92.3)
Current Smoker (*n* (%))	0 (0.0)
Excellent or Very Good Self-Rated Health (*n* (%))	10 (76.9)

Note: Results are presented as the mean ± standard deviation or frequency (percentage) as indicated.

**Table 3:** Time Spent in Each Activity Super- and Sub-Domain during the Telehealth Intervention.

Super-Domain	Sub-Domain	Baseline ^†^	Post-Intervention ^†^	Mean Change Minutes (95% CI)
Sleep	-	487 ± 38	489 ± 37	2 (−32, 36)
Physical Activity	-	22 ± 16	20 ± 29	−2 (−21, 17)
Work and Study	-	31 ± 29	122 ± 68	91 (45, 136)
-	Computer	26 ± 30	114 ± 72	88 (39, 137)
Transport	-	147 ± 151	77 ± 44	−70 (−167, 27)
-	Active transport	74 ± 39	38 ± 29	−36 (−62, −10)
-	Passive transport	73 ± 124	39 ± 32	−34 (−111, 43)
Quiet Time	-	133 ± 84	128 ± 72	−4 (−68, 59)
-	Reading	99 ± 95	108 ± 70	9 (−61, 78)
-	Other	33 ± 39	20 ± 20	−13 (−37, 11)
Self-Care	-	135 ± 32	127 ± 18	−9 (−32, 15)
-	Eating	78 ± 23	79 ± 11	1 (−69, 70)
-	Grooming	57 ± 16	48 ± 11	−9 (−22, 3)
Social	-	100 ± 80	101 ± 62	1 (−69, 70)
-	Communications	95 ± 74	80 ± 50	−15 (−82, 53)
-	Socializing	5 ± 11	21 ± 37	15 (−9, 40)
Chores	-	28 ± 100	207 ± 112	−1 (−101, 99)
-	Indoor chores	206 ± 99	191 ± 114	−15 (−114, 85)
-	Outdoor chores	2 ± 6	1 ± 29	13 (−5, 32)
Screen Time	-	135 ± 84	149 ± 106	14 (−75, 104)
-	Television	127 ± 83	149 ± 108	22 (−67, 111)
	Videogames	8 ± 17	0 ± 0	−8 (−18, 2)

^† ^Results are presented as the mean ± standard deviation min/day. Note: CI = confidence interval.

**Table 4:** Time Spent in Sedentary Behavior and Physical Activity during the Telehealth Intervention.

Variable	Baseline ^†^	Post-Intervention ^†^	Mean Change Time (95% CI)
*Daily Minutes*			
SB	688 ± 80	650 ± 82	−37 (−103, 27)
LPA	207 ± 57	201 ± 51	−6 (−50, 37)
MVPA	53 ± 27	55 ± 31	2 (−21, 26)
*Weekly Minutes*			
SB	4822 ± 560	4512 ± 643	−309 (−797, 179)
LPA	1454 ± 404	1406 ± 358	−47 (−357, 261)
MVPA	374 ± 195	387 ± 218	12 (−154, 180)

^†^ Results are presented as the mean ± standard deviation. Note: CI = confidence interval; LPA = light physical activity; MVPA = moderate-to-vigorous physical activity; SB = sedentary behavior.

## Data Availability

Data utilized in this study are not publicly available.
